# Trait mindfulness and sleep: Interactions between observing and nonreactivity in the association with sleep health

**DOI:** 10.1177/20551029221149282

**Published:** 2023-02-03

**Authors:** Elisabeth Bailin Xie, Ivan D. Sedov, Hangsel Sanguino, Makayla Freeman, Jeshna Kumari, Lianne Tomfohr-Madsen

**Affiliations:** 1 Department of Psychology, 2129 University of Calgary, Calgary, AB, Canada; 2 Department of Educational and Counselling Psychology, and Special Education, University of British Columbia, Vancouver, BC, Canada; 3 Faculty of Nursing, University of Calgary, Calgary, AB, Canada

**Keywords:** Mindfulness, Sleep, Health behaviour, Clinical health psychology, Health promotion

## Abstract

The current study investigated the associations between trait mindfulness and sleep health and examined the interactions between theoretically related mindfulness subscales. Participants (*n* = 162, M_age_ = 19.93) reported trait levels of mindfulness and sleep was assessed using questionnaires and actigraphy. Higher mindfulness scores in awareness, nonreactivity, and nonjudgment were associated with better sleep health. The associations between observing and sleep health were moderated by nonreactivity. Results indicate that observing is associated with better sleep health at higher levels of nonreactivity and worse sleep health at lower levels, helping to explain the often-contradictory findings between observing and health outcomes.

## Introduction

Trait or dispositional mindfulness has been defined as an individual’s tendency to maintain awareness of present moment experiences in a nonreactive and nonjudgmental way (Kabat-Zinn, 1990; Quaglia et al., 2016). Trait mindfulness is a multifaceted construct often assessed using the Five Facets of Mindfulness Questionnaire (FFMQ), which assesses skills in: (1) observing (i.e., noticing stimuli), (2) describing (i.e., labelling experiences), (3) acting with awareness (i.e., attending to the present moment), (4) nonjudging of inner experiences, and (5) nonreactivity to inner experiences (Baer et al., 2008; Truong et al., 2020). Higher trait mindfulness has been negatively associated with depression and positively linked to adaptive cognitive processes (Carpenter et al., 2019; Mattes, 2019; Tomlinson et al., 2017). A recent meta-analysis of the trait mindfulness literature concluded that, in addition to mental health variables, trait mindfulness is positively associated with an aggregated measure of “health behaviours”. Specifically, higher trait mindfulness across several facets was negatively associated with alcohol use and positively linked to more physical activity, healthier eating, and better sleep (Sala et al., 2020).

### Sleep health

The importance of sleep for the promotion of optimal physical and mental health is becoming increasingly recognized (Li et al., 2021; Freeman et al., 2020; João et al., 2018) and the promotion of adequate sleep to improve health, wellness and quality of life is included as a Healthy People 2020 public health priority (Healthy People, 2019). Identifying factors that promote or detract from optimal sleep has implications for public health (Hale et al., 2020; Hafner et al., 2017), and trait mindfulness may be one of these factors.

To comprehensively assess numerous correlated sleep variables that capture both subjective and objective information, the concept of “sleep health” has been proposed (Buysse, 2014). Sleep health is a multidimensional measure that includes variables that capture sleep duration, continuity, timing, perceived sleep quality and problems, and daytime sleepiness (Brindle et al., 2018). This multivariate construct is theorized to better capture the complex nature of sleep and has been associated with both physical and mental health outcomes (Dong et al., 2019). However, its association with facets of mindfulness has not been investigated.

### Facets of mindfulness and sleep

Previous investigations of the relationships between specific facets of mindfulness and sleep suggest that unique relationships may exist between individual factors of mindfulness and sleep. Acting with awareness, non-judging, and non-reactivity have been proposed to support engagement in health behaviours by equipping individuals to pay attention to their health behaviours and notice the impacts of their behaviours, while reducing their need to use health risk behaviours for emotion regulation (Sala et al., 2020). Specific facets of mindfulness linked to subjectively assessed sleep include describing, acting with awareness, nonjudgement, and nonreactivity, each of which have been associated with subjective sleep quality in the expected directions, such that higher mindfulness was associated with better sleep (Sala et al., 2020). Conversely, observing and describing are theorized to have a weaker relationship with health behaviours unless the other facets of mindfulness are present (Sala et al., 2020). When skills in other facets of mindfulness are low, it has been suggested that observing and describing may decrease engagement in health behaviours and increase engagement in health risk behaviours (Sala et al., 2020). Accordingly, meta-analysis has shown that simple associations between the observing scale and many health behaviours, including sleep, were not detected and, when associations did occur, they were in an unexpected direction; for example, higher observing was associated with higher substance use (Sala et al., 2020).

Several theoretical models have proposed that two facets of mindfulness are particularly important to cultivate together: (1) the cultivation of awareness of present moment experiences, including improving attention and concentration (e.g., notice physical surroundings, sensations, etc.) and (2) the development of a secondary reaction that includes an attitude of acceptance, nonjudgement and nonreactivity (Eisenlohr-Moul et al., 2012; Lindsay and Creswell, 2017). Without cultivation of the ability to notice present moment experiences in a nonjudgmental or nonreactive way, it is theorized that higher levels of observing may lead to distress and maladaptive coping (Eisenlohr-Moul et al., 2012). Indeed, in a recent study of undergraduate students, observing was associated with lower sleep quality as measured on the PSQI, while acting with awareness and nonjudgement were associated with higher sleep quality (Talley and Shelley-Tremblay, 2020).

Potential interactions in facets of mindfulness fit with theoretical models through which mindfulness interventions are thought to influence wellbeing – mainly through an increased awareness of present moment experiences coupled with the development of nonreactive acceptance (Kabat-Zinn, 1982, 1990). Several studies of health behaviours and outcomes have now shown interactions between the observing and nonreactivity scales on the FFMQ. In studies of undergraduate students, higher levels of observing have been shown to predict substance abuse when paired with low nonreactivity (Eisenlohr-Moul et al., 2012); however, observing was associated with lower levels of substance use and, in a separate study, of the proinflammatory cytokine interleukin-6, when nonreactivity was high (Eisenlohr-Moul et al., 2012; Tomfohr et al., 2014). Similar interactions have been found in studies of internalizing symptoms. In a sample of adults with diagnosed anxiety and depressive disorders, nonreactivity moderated the direct effect of observing on symptoms of depression (Desrosiers et al., 2014). More recently, this interaction was shown in a sample of adolescents at high risk for depression, such that nonreactivity moderated the effects of attention and awareness on internalizing symptoms (Stein et al., 2021). In studies of sleep, nonreactivity has been found to significantly moderate the relationship between observing and sleep quality, as measured using the PSQI, in a sample of healthy adults (Lau et al., 2017). However, more investigation into the relationship between facets of mindfulness and sleep health, particularly using comprehensive measures of sleep health, is needed.

### Aims of the current study

In this study, we investigated associations between individual facets of mindfulness (i.e., observing, describing, acting with awareness, nonjudging of inner experience and nonreactivity to inner experience) and sleep health. It was hypothesized that, in line with previous studies, there would be associations between unique facets of mindfulness and sleep health. We also hypothesized that observation of present moment experiences would interact with nonreactivity to predict sleep health, such that higher levels of observing would be associated with worse sleep in the context of low reactivity.

## Methods

### Participants

The sample included 174 undergraduate students from a large Canadian university. Participants were recruited through an opt-in university wide online research participation system. Participants ranged in age from 18 to 41 (*M* = 19.91, *SD* = 3.36). The sample included 90 women (51.7%) and 84 men (48.3%). Ethnicity of the sample was diverse with 40.2% of participants identifying as White, 40.1% as Asian, 4.6% as Black, 4.0% as Hispanic and 10.9% as another ethnic group. For the purposes of data analysis ethnicity was defined as white or other. The final sample included those who provided data to calculate the sleep score (*n* = 162). Sex of the final sample was roughly equivalent with 48% (*n* = 77) identifying as male and 52% (*n* = 85) identifying as female. Further sample characteristics are reported in Table 1. The study procedures were approved by the University of Calgary Conjoint Health Research Ethics Board and all participants provided informed consent.

**Table 1.** Characteristics of the sample.

	*M* (SD) or %	Valid *N*
Age	19.91 (3.36)	174
Sex (female)	51.7%	174
Ethnicity (white)	40.2%	174
BMI	23.85 (4.38)	172
Five facets of mindfulness		
Acting with awareness	25.36 (6.08)	
Nonreactivity	21.32 (4.65)	
Nonjudgement	24.55 (6.49)	
Describe	26.66 (5.77)	
Observe	26.27 (5.34)	
Sleep health	2.46 (1.25)	162

Note: BMI = body mass index.

### Procedure

Eligible participants completed a battery of questionnaires that collected self-reported demographic information (i.e. age, sex, and ethnicity), socioeconomic status, subjective sleep quality and depressive symptoms. Participants were also asked to wear a watch-like sleep monitoring device (an Actiwatch) for 72 hours in order to capture three nights of objective sleep data. A handwritten sleep diary was provided to every participant to complete (e.g., reporting when they went to bed, how long it took them to fall asleep, etc.), which coincided with wearing the actigrapher. The sleep diaries were used to score and validate actigraphy data and to corroborate actigraphy data that was unclear.

### Measures

#### Five Facets of Mindfulness Questionnaire (FFMQ; Baer et al., 2008).

Participants completed the 39-item FFMQ, which assesses five distinct aspects of mindfulness. The five factors of the FFMQ include: observing, describing, acting with awareness, nonjudging of inner experience and nonreactivity to inner experience. Participants rated the degree to which each of these items applied to them using a 5-point Likert-type scale (1 = *never or very rarely true*, 5 = *almost always or always true*). The FFMQ has been documented to have good internal consistency in samples of undergraduate students, with α coefficients ranging from .75 to .91. In our study, the FFMQ Cronbach’s α were all within an acceptable range [*observing* (α = .74), *describing* (α = .85), *acting with awareness* (α = .88)*, nonjudging of inner experience* (α = .88), *nonreactivity to inner experience* (α = .75)].

#### Pittsburgh Sleep Quality Index (PSQI).

Subjective sleep quality was assessed using the 19-item self-administered PSQI (Buysse et al., 1989). The PSQI evaluates retrospective sleep quality and disturbances within the past month. It measures seven components of sleep: subjective sleep quality, sleep latency, sleep duration, habitual sleep efficiency, sleep disturbances, use of sleeping medication, and daytime dysfunction. Summing each sub-scale creates a global PSQI score. Global PSQI scores range from 0 to 21, with higher scores indicating lower sleep quality and a more severe sleep disturbance. Scores greater than 5 on the PSQI have traditionally been used to define poor sleep quality. The total PSQI had good internal consistency with a Cronbach’s α = .83. Items 6 (sleep quality) and 7 (daytime sleepiness) from this measure were included in the calculation of the sleep health score in the current study.

#### Actigraphy

Actigraphy sleep data were collected using Philips Respironics Actiwatch 2 monitors and analyzed using Philips Respironics software. Actiwatch 2 monitors provide estimates of sleep onset, sleep latency, wake after sleep onset (WASO), number and length of awakenings, sleep duration, and sleep efficiency. Sleep latency is described as the length of time it takes for someone to fall asleep. The actiwatch software measures this by calculating the time between ‘lights off’ to the first 3 minutes of sleep. WASO refers to the number of minutes awake between sleep onset and time of final waking. Sleep efficiency is the proportion of the estimated sleep periods spent asleep. Actigraphy is a cost effective, reliable, and convenient (acceptable by participants) objective measure of sleep; importantly, it is a non-intrusive monitor of daily sleep patterns, as it is convenient for home/natural sleep setting use, requiring no overnight lab stays or at home set ups (Martin and Hakim, 2011; McCall and McCall, 2012). The Actiwatch 2 monitors were provided to participants after the completion of in-person questionnaires. Means for the data were calculated for each of the three-night’s sleep parameter and described using standard deviations (Rijlaarsdam et al., 2014).

#### Sleep health

The sleep health variable was created following the recommendations from Brindle et al. (2018); however, the *Sleep Timing* component was further adjusted using guidelines from Tonetti, Fabbri and Natale (2008). The proposed measure of sleep health included six dimensions of sleep; Sleep Regularity, Sleep Satisfaction, Sleep Timing, Sleep Efficiency, Sleep Duration, and Daytime Sleepiness*.* Sleep regularity, timing, efficiency, and duration were derived from data collected by Actigraphy. Further, a similar adapted version of a sleep health variable which included sleep regularity, satisfaction, alertness, timing, efficiency, and duration has been shown to have high internal validity with an overall reliability coefficient of .85 (Becker et al., 2018b). Each component of the sleep health variable was dichotomized as 0 or 1, with higher scores indicating high overall quality of sleep. Scores for the sleep health variable ranged from 0 to 6.

Sleep Regularity*,* defined as the fluctuation in timing of one’s sleep midpoint, was calculated by first establishing the sleep midpoint between bedtime and rise time for each day of the 3-days of assessment. Next, midpoint sleep fluctuations across the 3 days (i.e., SD for midpoints) were identified and a 1-hour cut-off was used to measure whether participants had irregular or regular sleep. Participants with midpoint sleep fluctuations greater than 1-hour were given a value of 0, while participants with sleep fluctuation less than 1-hour received scores of 1. This cut-off was selected based on previous literature in adolescents (Dong et al., 2019). In our sample, the mean SD for midpoint sleep was 2 hours and 38 minutes.

Sleep Satisfaction refers to the subjective evaluation of one’s sleep. Participants sleep satisfaction was derived from item 6 on the PSQI. The question asks participants “*During the past month, how would you rate your sleep quality overall”.* Participants with scores of 2 “fairly bad*”* or 3 “very *bad*” on item 6 of the PSQI were given a value of 0 while those with scores of 0 “very *good”* or 1 “fairly *good”* on the same item were given a value of 1.

Sleep Timing*,* defined as appropriate timing of sleep midpoint within a 24-hour day (Buysse, 2014), was calculated following established recommendations (Tonetti et al., 2008). We used the Tonetti et al. (2008) ideal midpoint range for our range, which allowed us to systematically account for sex differences and age of participants. Scores of 0 were given to participants with an average midpoint outside the set range while those with an average midpoint falling within the set range received a score of 1.

Sleep efficiency is defined as the percentage of time a person is in bed and asleep versus the total amount of time spent in bed. Derived using Actigraphy, sleep efficiency was calculated by dividing total sleep time (TST) by total time in bed (TIB) and multiplying by 100. Participants with average sleep efficiency ≥ 85% received a score of 1 (i.e., good sleep efficiency), while scores of 0 were given to participants with sleep efficiency <85%.

Sleep duration is defined as the total amount of time spent asleep. This was averaged across the 3-day Actigraphy data. Cut-offs were set based on prior literature recommendations. Sleep that was less than 7 hours (420 minutes) or more than 9 hours (540 minutes) was scored as 0 while 1 was indicative of a ‘healthy’ amount of sleep; between 7 and 9 hours (Hirshkowitz et al., 2015).

Daytime Sleepiness is a rating of how alert and wakeful an individual is during the day. Item number 7 of the PSQI was used to operationalize the sleepiness component of the sleep health variable. Participants received a 1 if they answered 0 “*not during the past month”* or 1 “*Less than once a week”* or a 0 if they answered 2 “*Once or twice a week”* or 3 *“Three or more times a week.”*

#### Anthropometric measurement

Participants’ height and weight was measured by a research-assistant using the Health-O-Meter mechanical beam scale with height rod for the purpose of calculating the BMI covariate. Body weight was recorded in kg and height in meters. BMI was then calculated by dividing weight (kg) by squared height (m^2^).

#### Covariates

Covariates included sex, ethnicity*,* age, and BMI. Covariates were decided a-priori and theoretically justified based on previous literature suggesting that sex (Madrid-Valero et al., 2017), ethnicity (Eastman et al., 2015; Stepnowsky et al., 2003), age (Mander et al., 2017), and BMI (Gonnissen et al., 2013) are associated with sleep health.

### Statistical analyses

Statistical analyses were performed using SPSS (Version 26.0) statistical software package (IBM Corp., 2019). Relationships between mindfulness, sleep health and covariates were explored using correlational analyses. Ethnicity was dichotomously coded as White = 1 versus “other” = 0 for the purposes of the analyses. Gender was coded as female = 0 and male = 1. Hierarchical regression analysis was used to investigate relationships between facets of mindfulness and sleep health. First, we examined correlations in unadjusted models. Next, in regression models we adjusted for demographic covariates (age, ethnicity, sex, BMI).

Hypothesized moderation was testing using the PROCESS macro for SPSS (Hayes, 2013). The Johnson-Neyman technique and tests of regions of significance simple slopes were examined to determine the points at which facets of mindfulness had a significant conditional effect in the prediction of sleep health.

#### Missing data

Participant data were included if there was information available to be able to calculate both sleep health scores. Missing values analysis in SPSS 26 was conducted to explore patterns of missingness. Little’s MCAR test was conducted to determine whether the values were missing completely at random (MCAR). The results of the missing data analysis indicated that out of BMI, age, sleep health, and the FFMQ, only the sleep health variable had more than 5% missing data (6.9%). The results of the Little’s MCAR test indicated that the data were missing completely at random (Chi-square = 5.38, *p* = .98).

## Results

### Bivariate correlations

Table 2 shows correlations among study variables. Sleep health was positively associated with acting with awareness (AWA), nonreactivity, and nonjudgement.

**Table 2.** Correlations between sleep, facets of mindfulness, and covariates.

	Sleep health	AWS	Nonreactivity	Nonjudgment	Describe	Observe	Race	BMI	Sex
Sleep health	—								
Act with awareness	.267**	—							
Nonreactivity	.171*	.178*	—						
Nonjudgement	.262**	.377**	.095	—					
Describe	.083	.478**	.271**	.113	—				
Observe	−0.039	.056	.267**	−0.321**	.230**	—			
Race	−0.065	−0.043	.076	−0.025	−0.044	−0.011	—		
BMI	.024	−0.052	.074	−0.109	.089	−0.099	−0.079	—	
Sex	.002	−0.106	−0.349**	.025	−0.053	.012	.129	−0.238*	—
Age	.084	.069	−0.014	.024	.052	−0.094	−0.19*	.282**	−0.282**

Note: AWA = Acting with Awareness; BMI = Body Mass Index. Note: *p < .05; **p < .01

### Relationships between facets of mindfulness and sleep health

Unadjusted (see Supplemental Table 1) and adjusted models produced similar results so only the adjusted models are discussed here. Results of adjusted regression analyses are presented in Table 3. After adjusting for age, ethnicity, BMI and sex, acting with awareness, nonreactivity and nonjudgment each remained associated with sleep health in the expected directions.

**Table 3.** Linear regression analyses showing Five Facets of Mindfulness variables predicting Sleep Variables.

Outcome: Sleep health					
Predictor	*β*	*SE*	*p*	ΔR^*2*^	*B 95% CI [LL, UL]*
AWA	0.054	0.016	<0.001*	.069	[.023, .086]
Nonreactivity	0.057	0.022	.013*	.039	[.012, .101]
Nonjudgement	0.050	0.015	<0.001*	.068	[.021, .080]
Describe	0.016	0.017	.353	.006	[−.018, .049]
Observe	−0.007	0.019	.691	.001	[−.045, .030]

Note; AWA = Acting with Awareness.

* Indicates statistically significant p-value

Model is adjusted for age, body mass index, ethnicity, and sex, LL and UL indicate the lower and upper limits of a confidence interval, respectively

**Figure 1.**
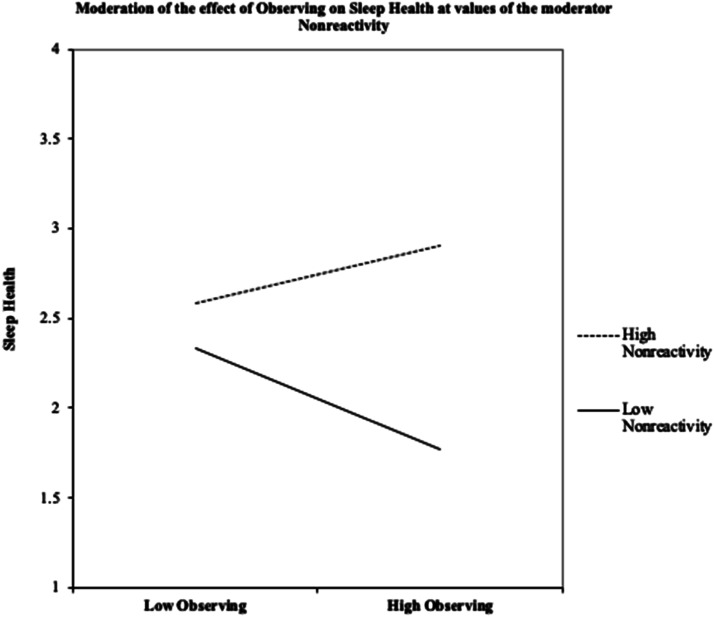
Standardized conditional moderation effect of nonreactivity on the relationship between observing and sleep health.

Next, regression analyses were conducted to assess if associations between observation of present moment experiences and nonreactivity were predictive of sleep health. The interaction was significant for the prediction of sleep health (β = -.0087, *SE* = .0031, *p* = .0064, 95% CI [0.0025–.0149], ΔR^2^ = 0.045) such that observing was associated with better sleep health but only in the context of higher nonreactivity. See Figure 1.

**Figure 2.**
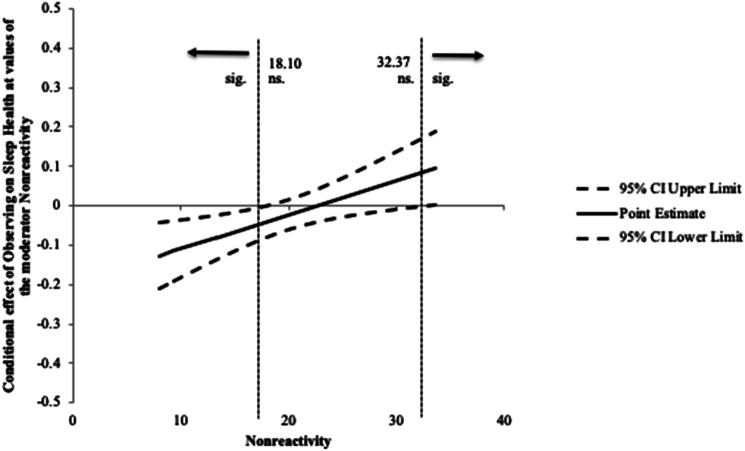
Johnson-Neyman confidence limits for the conditional moderation effect of nonreactivity on the relationship between observing and sleep health.

To interpret the interactions, Johnson-Neyman regions of significance tests were conducted. Results showed that the point of transition between a statistically significant and nonsignificant effect of nonreactivity was below 18.10 (26.88% of the sample was below) and above 32.37 (0.63% of the sample was above). This means the effect of nonreactivity on sleep health was significant when participants had a score below 18.10, such that higher observe scores below this number were associated with worse sleep health. In the range above 32.37 on nonreactivity the observe scores were associated with better sleep health. See Figure 2.

## Discussion

The purpose of this paper was to examine if the individual facets of mindfulness (i.e., observing, describing, acting with awareness, nonjudging of inner experience and nonreactivity to inner experience) are associated with sleep health and to test whether the effect of observing on sleep health differs depending on levels of nonreactivity. The results show that observing is related to sleep health but whether observing is positively or negatively associated with sleep health is dependent on the ability to respond nonreactively to present moment experiences. This paper adds to a growing literature showing interactions between observing and nonreactivity in the prediction of mental and physical health outcomes, including sleep (Desrosiers et al., 2014; Eisenlohr-Moul et al., 2012; Stein et al., 2021; Tomfohr et al., 2014; Lau et al., 2017). The interactions between observing and nonreactivity help to explain the often inconsistent or contradictory findings between observing and physical and mental health variables (Carpenter et al., 2019; Brown et al., 2015; Curtiss and Klemanski, 2014). Specifically, our finding suggests that one reason why observing has been found to have different effects on health outcomes (i.e., sleep) is because the association between observing and sleep health differs based on one’s levels of nonreactivity.

Meta-analyses show that mindfulness-based intervention (MBIs) are associated with improvements in subjective sleep quality when compared to control groups, However, the mechanisms through which the changes occur are not entirely clear (Rusch et al., 2019; Wang et al., 2020). Ong et al. (2012) hypothesize that training in mindfulness improves sleep both through increased awareness of mental and physical states that are associated with insomnia and also through the development of an adaptive stance towards these symptoms including, cognitive flexibility, more balance appraisals of sleep problems and equanimity or nonreactivity when poor sleep is encountered (Ong et al., 2012). Results from this paper appear to support these proposed mechanisms suggesting that increased observing coupled with a less reactive or more adaptive stance may lead to better sleep. Additionally, a study of depressed patients suggested that interactions between observing and nonreactivity were associated with cognitive emotion regulation strategies, including worry, rumination and reappraisal (Desrosiers et al., 2014). The authors of this study suggest that observing paired with nonreactivity may allow for individuals to avoid unproductive cognitive processing (i.e., worry, rumination; Desrosiers et al., 2014). Engaging in perseverative cognition such as worry and rumination is strongly associated with worse sleep (Clancy et al., 2020); a potential mechanism through which mindfulness is associated with better sleep may be through a reduction of these processes.

Structured mindfulness-based treatment programs typically offer comprehensive treatments that address all facets of mindful attention through the use of diverse formal and informal practices (Kabat-Zinn, 1990, 2003). Drawn from a broader religious and philosophical background, many of the mindfulness interventions most commonly delivered in Western contexts follow a set of scripts delivered, sometimes with little consideration of the broader traditions and context from which they were drawn (Grossman and Van Dam, 2011). The findings raise questions about the importance of actively increasing nonreactivity in the context of increasing observing in mindfulness interventions. Although these are core components of many mainstream mindfulness treatments, the importance and sequencing of increasing nonreactivity while also strengthening attention has received very little attention in the literature. Although rarely investigated, some studies are beginning to look at the way facets of mindfulness change in relation to one another in the context of mindfulness interventions. For example, in a study comparing mindfulness to a health information control group, there was no difference in changes in FFMQ scores between the two interventions; however, the groups did differ in their associations between observing, acting with awareness, and nonjudging, suggesting that changes in mean scores on the FFMQ may not be accurately capturing the complex changes in mindset that occur in the context of the mindfulness interventions (Goldberg et al., 2016). Clinicians employing mindfulness practices to promote better sleep health in a less structured format with their clients should be considerate of the need to foster the nonjudgment and nonreactivity quality of attention simultaneously with the more sensation focused qualities.

Mindfulness is beneficial for mental and physical health. For instance, mindfulness-based interventions (MBIs) have been shown to reduce psychological distress and improve a variety of physiological measures including aspects of immune system activity and longer telomere lengths (Black and Slavich, 2016; Goldberg et al., 2018; Hofmann et al., 2010; Schutte et al., 2020). Sleep is critically important for physical and mental health (João et al., 2018; Freeman et al., 2020; Li et al., 2021; Healthy People, 2019; Lange et al., 2003; Dew et al., 2003). Thus, potential improvements in sleep is another pathway through which mindfulness may contribute to improvements in overall health.

### Limitations and future directions

The findings must be interpreted considering several limitations. The data was cross sectional in nature and the findings bear replication in longitudinal design or randomized controlled trials where examination of mechanisms is possible. This research was also limited by not being pre-registered, which should be completed in future studies. Furthermore, the undergraduate sample recruited may not generalize to other populations with more clinically significant sleep or mood concerns. However, the study of sleep among undergraduate students is valuable as this group frequently reports sleep difficulties due to physiological and developmental changes as well as school, work, and social demands (Becker et al., 2018a, 2018b; Owens et al., 2017). Among undergraduates, better sleep quality is linked to better academic and mental health outcomes (Orzech et al., 2011; Becker et al., 2018a). The high prevalence and negative consequences of sleep concerns in this population makes this an important group to target for sleep interventions. Finally, although sleep was not assessed via gold standard polysomnography, a significant strength of the current paper was the incorporation of objective assessments of sleep data and the creation of a sleep health variable, as it allowed us to use a more comprehensive sleep measure.

Several important research questions emerge from this study. First, the current findings should be replicated using a longitudinal sample to test whether the effects are similar temporally. Relatedly, micro-longitudinal studies examining the effects of daily changes in facets of mindfulness on sleep health would be highly beneficial. Second, replication of the current findings with individuals from clinical samples to whom mindfulness-based interventions are often delivered is an important next step. Third, randomized controlled trials of mindfulness-based interventions on sleep health with increases in nonreactivity as possible mediators of treatment effect should be conducted. Future studies should use larger sample sizes to ensure the study is adequately powered to detect the effects of interest. Finally, researchers have yet to establish whether certain mindfulness techniques are more effective at increasing various facets of mindfulness. Exploring whether certain exercises are especially well-suited to increasing clients’ nonreactivity may be helpful to clinicians.

## Supplemental Material

Supplemental Material - Trait mindfulness and sleep: Interactions between observing and nonreactivity in the association with sleep health Click here for additional data file. Supplemental Material for Trait mindfulness and sleep: Interactions between observing and nonreactivity in the association with sleep health by Elisabeth Bailin Xie, Ivan D SedovHangsel Sanguino, Makayla Freeman, Jeshna Kumari, and Lianne Tomfohr-Madsen.
