# Supplementary material for: Trait mindfulness and sleep: Interactions between observing and nonreactivity in the association with sleep health
Source: Health Psychol Open. 2023 Feb 3;10(1):20551029221149282. doi: 10.1177/20551029221149282 (PMC9900671; doi:10.1177/20551029221149282)
Supplement: Supplemental Material - Trait mindfulness and sleep: Interactions between observing and nonreactivity in the association with sleep health [file sj-pdf-1-hpo-10.1177_20551029221149282.pdf]

Supplemental Table 1

Linear regression analyses showing Five Facets of Mindfulness variables predicting Sleep Variables – Unadjusted model

Outcome: Sleep Health					
Predictor	*β*	*SE*	*p*	ΔR^2	*B 95% CI [LL, UL]*
AWA	0.054	0.016	< .001*	.071	[.024, .085]
Nonreactivity	0.046	0.021	.029*	.029	[.005, .086]
Nonjudgement	0.050	0.015	< .001*	.069	[.021, .079]
Describe	0.018	0.017	.295	.007	[-.015, .050]
Observe	-0.009	0.018	.626	.001	[-.045, .027]

Note; AWA = Acting with Awareness
* Indicates statistically significant p-value
LL and UL indicate the lower and upper limits of a confidence interval, respectively
